# 

**Published:** 1888-02-25

**Authors:** 


### SPECIAL NOTICE TO NEWSAGENTS, ADVERTISERS, AND THE TRADE.

### CHANGE OF OFFICE AND PUBLISHER.

NOTICE is hereby given, that the Office of THE HOSPITAL will henceforth be at 38, Old Jewry (Cheapside end), E.C., to which address all communications should be sent. Mr. A. Doig has been appointed Advertising Agent, and Mr. J. H. S. Hanning, Manager. All Cheques and Post Office Orders should be made payable to J. H. S. Hanning, 38, Old Jewry, E.C., crossed Lloyds, Barnetts, and Bosanquets Bank (Limited).

### QUESTIONS AND ANWSERS.

### CONTINENTAL NURSING INSTITUTIONS.

Can any reader answer the following question by Nurse Johnson:—

Would you be so very kind as to tell me of *some Continental Institutions for Nurses?* I shall be leaving the Kent Institute in the summer, and am anxious to get remunerative work as a monthly nurse abroad—in Italy, Germany, or South of France. I enclose stamped envelope. If any one should send the addresses of some such institutions I should be very grateful.

C. Frost is referred to Vol. III., Nos. 68 and 70 (January 14th and 28th, 1888) for information as to the use of kola in alcoholism.

Miss J. Nelson is thanked for her letter.—Ed. *T. H.*

### Amusements with Profit for all Ages.

**Rules.**—All answers must be addressed to the Prize Editor, The Hospital, 38, Old Jewry, Cheapside, London, E.C., not later than the *first* post on Thursday next. The full name and address must in all cases be given, but a *nom de plume* may be added for publication. Only one side of the paper must be written on.

### FOR MEN AND WOMEN.

**Rule.**—Competitors can enter for *all* quarterly competitions, but no competitor may take more than *two quarterly prizes* during the year.

### Acrostic Competition.

Second quarter commences February 25th, 1888; ends May 19th, 1888.

A PRIZE OF ONE GUINEA will be given to the competitor who gains the highest number of marks for best original acrostics during the ensuing quarter. All acrostics sent in for this competition will be credited according to merit, twelve marks being the highest score given for one acrostic.

A PRIZE of HALF-A-GUINEA to be given to the competitor who gains the highest score for solution of acrostics set. *One mark* only will be given to the competitors who solve all the lights of each acrostic.

Exception will be made in the case of *quotation acrostics*, when *two marks* will be given, *one* for the correct solution of all *lights*, and *one* mark for the *source* of the quotations.

These competitions to alternate weekly.

Credit will be given this week for solution of—

No. 1. Double Acrostic.

In vision dark my *primals* show
A void and formless mass.
That mass my *finals* swift endue
With loveliness, where beauties new
Past beauties still surpass.

Nature and Art once made
A compact to produce
A portrait: she the layers worked in
Of pigments for art's use—
A gem their canvas was;
Art work'd out Nature's hints,
And raised upon a rich dark ground
A face in pearly tints.

To Charles the Saracens—
The Scots to Edward gave
A name that proved these conquerors
Irresistible as brave.

A prince once possest of a magical tent,
Which he carried about in his pocket;
Yet at will could spread out to such an extent,
That 'twould take a whole army to stock it.

The while her stalwart paramour
Her distaff idly plied,
She reign'd great lady paramount
Array'd in lion's hide.

Ye dancing creature
Of hybrid race,
Your human features
A brute deface. Pasteur.

Results of No. 6 Original Double Acrostic.—Pasteur (11), Brownie (11), Patience (10), Padre (9), Madge (9), Mater (9).

*Results of First Quarterly Acrostic Competition will be Published next week.*

(A) THE WORD COMPETITION.

We shall give the newspapers and periodicals for dissection during this quarter, that for this, the *fourth* week of the quarter, being "Academy."

Observe.—Proper names, abbreviations, foreign words, words of less than four letters, and repetitions are barred; plurals, past and present participles, or verbs are allowed. Nuttall's *Standard* dictionary only to be used.

N.B.—All words sent in must be arranged *alphabetically*, with correct total affixed.

### Results of Second Week.

*Observer.*—Gretta (54), Patience (53), Dollie (53), E. E. (52), Condorcet (50), Ellice (49), Brisbane (38), Ninny (38), Lightowlers (38), Rowdy Corner (38) Dunce (37), Lancashire Witch (37), Lodge (36), Lauriston (35), Qui vive (34), Pigeon (34), Isabel (33), Susan Nipper (33), Sym (32), Nelle (30)

(B) THE LITERARY COMPETITION.

Some people hate puzzles of all kinds, including word competitions, but they are fond of correspondence, and many sisters and mothers are continually receiving and sending letters to and from members of the family who are abroad in India or the Colonies. The constant coming and going between England and other portions of the British Empire make it a matter of interest and importance to know something about the life, the climate, the amusements, the adventures, the clothing, and the surroundings of those who seek a livelihood beyond the seas. We shall therefore try to find space for the publication of selected letters from abroad, which may be sent by any of our readers who have friends and relations in foreign lands. We shall give marks for these letters as well as for the word competitions which will count equally for these prizes, so that anyone may enter for both or either, the prizes to be given to those who get the highest number of marks.

### Letters from Across the Sea.

*Gretta* forwards the following letter from Montgomery, Ala., U.S.A., January, 1888.

An American Quack, &c.

We have lately been enduring the most miserable weather, bleak, raining, and at times cold; then warm again. After rain all night, this morning we have a gale of wind; though I am thankful to say the sun is now shining. I think we are coming in for a share of the terrible western and northern blizzards. We have had no snow so far this winter. I have been a great sufferer from neuralgia over my eyes, and have found no permanent cure. We have quite a curiosity in the shape of a California man exhibiting in town now. His name is "Yellowstone Kit;" he is worth five million dollars, and has made it all from selling a "pad," which adjusted to the stomach, is supposed to cure nearly all bodily ailments. He claims it to be made of a Japanese herb, which he went all the way to Japan to hunt for. He gives free entertainments, having a Japanese lady and gentleman who perform tricks, and the "most wonderful contortionists" many have ever seen, the latter actually dislocating his neck and replacing it again, the Japs swallowing whole eggs, &c. He has also a fine band, and wears $10,000 worth of diamonds on his coat. Every Friday he gives packages of food and clothing away to the needy. He sells these pads at $1 if you buy one in a limited time of two minutes; after that you must pay $2 for them; he has sold thousands here. H—— bought me one during the two minutes. I tried it for neuralgia, put it on my head, and it relieved me. He was well guarded with policemen the night we attended and his detectives all round town; the negroes are wild over him. We saw him mesmerise a dozen men and women for rheumatism, deafness, and toothache.

To-morrow a set of "wax works" are to be exhibited next door to us; the old couple who have them in charge say that they came from "Madame Tussaud's" but I am sure they never saw each other. (The writer is an American lady, and though she has been to "Tussauds," does not understand Madame is dead.) In December we had two tremendous conflagrations, the first the Montgomery flour mills. We were rather interested in that, for it was such a beautiful sight, and happened early in the evening. The second were three large grocery stores, just diagonally across from where we are boarding; it was very awful. It broke out just at midnight, when we were all in a deep sleep. I am easily awakened through being so accustomed to getting up to the children, so I heard the cry of "Fire" before the bell rang, and bounded up in a second. At once I smelt the smoke, and knew it was around us. I shook and shook H——, and finally aroused him. "Oh!" he said, "the fire's not here; what's the use?" But when he discovered the terrible smoke he put on his clothes and rushed across the street. By this time the fire had gained headway, and nothing could be done (the engines were pumping for two days afterwards) The falling walls, gunpowder explosions, and the oil made it terrible. My sister, who was just recovering from a spell of fever, was very hysterical, whereupon I gave her a good scolding. The sparks were flying all over our house, so we just dressed, not knowing what time we might have to vacate. Sure enough, the dreaded hour came. H—— had just gone down to the office for a few minutes, when the fireman rushed in and told us to vacate. The proprietors were down the street looking at the fire. No one in the house but my sister and her child and I and my three little ones; so I first bundled up the girl in a blanket and gave her to a white man I begged to help me in the street, then I got H ——, jun., in a blanket and got a negro to take him, and my baby I gave to a negro woman; so each one was carried to a different house, and I followed bareheaded till I found them, and then told them to rest quiet till I came back, as I wanted to see to my things. On my road back to the house I met H——, who was much alarmed when he heard the news about our house, and he came running back, to find his little flock all gone! but our house did not catch, and we were indeed lucky to escape.

### THE CHILDREN'S CORNER.

PRIZES.
FOR MOST CORRECT ANSWERS TO PUZZLES.
This Commenced October 1st, 1887.
One Pound a Month.
A PRIZE OF THREE GUINEAS
to the Competitor who shall have sent in the largest number of correct or best answers during the twelve months.

### Puzzles—Fourth Week.

No. 13. Geographical Acrostic.—1. An Arabian seaport. 2 A town in South Wales, famous for its iron works and docks. 3. A province of Brazil. 4. A Prussian town celebrated for its fortress. 5. An island east of Madagascar. 6. Province of Spain. Primals and finals, give the name of an African lake.

No. 14. Arithmetical Puzzle.—*I* am worn alike by old and young, my name is formed by three letters of the alphabet. The first letter's place is three times that of the second; the third is five times that of the first + 1; and the sum of all three letters' places is twenty.

No. 15. Charade.

Shepherds on my *First* in golden ages lay,
To hear my *Second* carolled from each spray:
My *Whole* they never knew, nor should we know
If we of modern days, lived in an age of gold.

No. 16—Enigma.

When no sound or voice is heard,
No lips to utter but a word,
Then I exist and not before,
Now name me—I exist no more.

### Results of Second Week Puzzles.

No. 5.—Buried Rivers.—1. Wye 2. Thames. 3. Ouse. 4. Severn.

No. 6.—Charade.—Parson-age=Parsonage.

No. 7.—Biographical Acrostic.

M odel L
I ndig O
L anter N
T orpi D
O tt O
N otatio N

No. 8.—Art-i-choke=Artichoke.

*All right*—None. *Three right*—Robinson, Druce, Alert, Spider, Plummy Fluff, Excelsior, Oswald H., Moina, A. C. K. *Two right*—Poodle, Alsie, A. G. S., McCulloch, Mount Edgecumbe. Jerusalem, Scrooge, Betty Burke, Squeaker.

All competitors who send in papers must strictly abide by the following

### Conditions.

I. Every paper sent in must be clearly written, and one side only must be used. All competitors must mark at the head of their papers the number of their competition.

II. Each paper must be signed by the author with his or her real name and address. A *nom de plume* may be added, if the writer does not desire to be referred to by us by his real name. In the case of all prize-winners, however, the real name and address will be published.

III. All communications relating to prize competitions should be addressed to "The Prize Editor, The Hospital, 38, Old Jewry, London, E.C." Competitors are requested not to include in letters, etc., to the Prize Editor communications on matters of other business. All letters must be fully prepaid.

IV. The Prize Editor of "The Hospital" is the sole judge of the competitions, and his decision is in all cases final and without appeal.

V. The Prize Editor reserves the right to publish any paper sent in, whether it receives a prize or not. He does not hold himself responsible for any MSS. sent, nor can he undertake to return rejected competitions.

**Notice to all Correspondents.**—N.B.—All letters referring to this page, which do not arrive at 38, Old Jewry, Cheapside, London, E.C., by the *first post on Thursdays*, and are not addressed PRIZE EDITOR, will in future be disqualified and disregarded.

No one will be permitted to win the Monthly or Quarterly Prizes twice in any one year, the annual prizes being offered especially to prevent this.

